# *De novo* transcriptome assembly and analysis of *Sf*21 cells using illumina paired end sequencing

**DOI:** 10.1186/s13062-015-0072-7

**Published:** 2015-08-20

**Authors:** Pavan Kumar Kakumani, Rohit Shukla, Vivek N. Todur, Pawan Malhotra, Sunil K. Mukherjee, Raj K. Bhatnagar

**Affiliations:** International Centre for Genetic Engineering and Biotechnology, Aruna Asaf Ali Marg, New Delhi, 110067 India; Bionivid Technology Pvt. Ltd., 401, 4 AB Cross, 1st Main, Kasturi Nagar, NGEF East, Bangalore, 560043 India; Present address: Department of Genetics, University of Delhi South Campus, Benito Juarez Road, New Delhi, 110021 India

**Keywords:** Army worm, Insect, Pest, Sf21 cells, *Spodoptera frugiperda*

## Abstract

**Abstract:**

*Spodoptera* is an important polyphagous agricultural insect pest in the tropical world. The genomic details are limited to understand the pest biology at molecular level. In the present study, we sequenced and assembled the transcriptome from Sf21 cells into a non redundant set of 24,038 contigs of ~ 47.38 Mb in size. A total of 26,390 unigenes were identified from the assembled transcripts and their annotation revealed the prevalent protein domains in Sf21 cells. The present study would provide a resource for gene discovery and development of functional molecular markers to understand the biology of *S. frugiperda.*

**Reviewers:**

This article was reviewed by Dr. Thiago Motta Venancio and Prof. Michael Gray.

**Electronic supplementary material:**

The online version of this article (doi:10.1186/s13062-015-0072-7) contains supplementary material, which is available to authorized users.

## Findings

The fall armyworm, *Spodoptera frugiperda* (*S. frugiperda*) is classified under Lepidoptera, the second largest order of insects which includes some of the most destructive agricultural pests. Considering the agricultural and economical importance of *S. frugiperda*, our group generated the draft assembly of genomic DNA from Sf21 cells, a cell line derived from the ovary of *S. fruigperda* [1]*.* To take the genome sequence application further, we have integrated available EST data of *Spodoptera* and complemented with transcriptomic data to generate more comprehensive information of Sf21 cells.

**Fig. 1:**
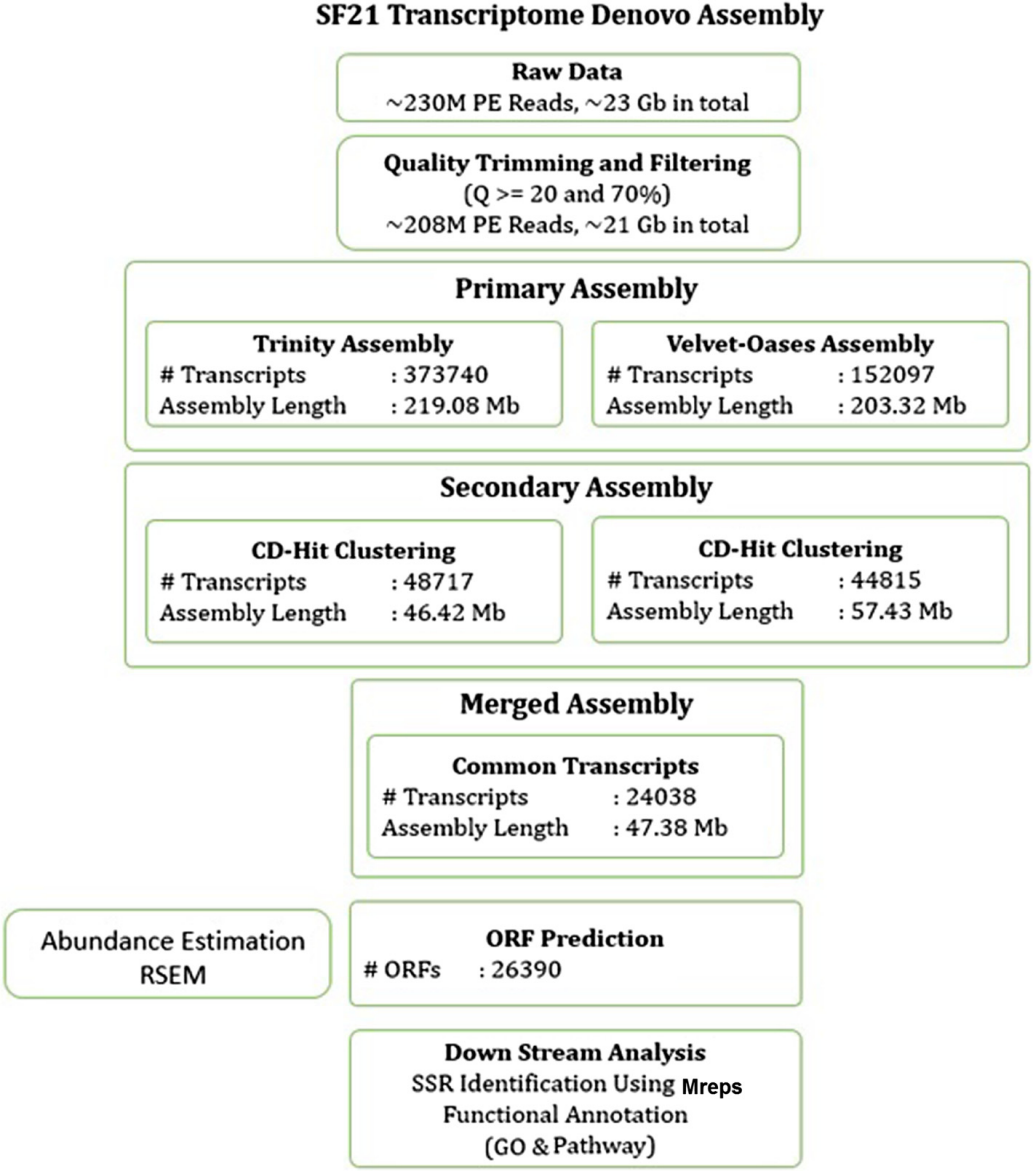
The flow chart of data analysis: display of the main steps and volumes of raw, pre processed data and number of identified unigenes

To characterize the transcriptome of Sf21 cells, total RNA isolated from Sf21 monolayer was used to prepare the library and subjected to high throughput sequencing on Illumina HiSeq 2000 platform. The comprehensive approach followed for the assembly and annotation of the transcriptome is presented in Fig. 1. A total of ~ 23Gb data (~230 M reads) was obtained from the sequencing and the quality control resulted in ~ 208 Million HQ paired end reads. The high quality reads were used to generate a primary assembly using the tools, Trinity [2] and Velvet-Oasis [3], independently. The Trinity assembly resulted in a total of 373,740 contigs with total length of 219.08 Mb. Similarly, the Velvet-Oasis assembly resulted in a total of 152,097 contigs of size 203.32 Mb. Next, to generate a non-redundant full length transcriptome, the homologous contigs were clustered using CD-HIT-EST (v4.6.1) [4], resulting in a total of 48,717 transcripts (46.42 Mb) and 44,815 transcripts (57.43 Mb) from the Trinity and the Velvet-Oasis assemblies respectively (see Additional file 1). Further, the clustered transcripts were merged to achieve a final assembly of 24,038 non redundant contigs of total length, 47.38 Mb at an N50 of 3.4Kb, while the mean and maximum length of the contigs are 1.97Kb, 28.91Kb respectively (see Additional file 2A). In addition, the unigenes encoding proteins were identified from the contigs using EMBOSS [5, 6]. The analysis resulted in a total of 86,059 short open reading frames which were further clustered to achieve a total of 26,390 unigenes with a minimum length of 300 bp, while the maximum and mean length of unigenes are 25.86Kb and 816.8 bases. The length wise distribution of the unigenes is presented in Additional file 3A, indicating the trancriptome with broad range of transcripts. To evaluate relative quality of the assembly, we performed BLAT analysis with 70 % coverage and identity by comparing the transcriptome data against the genome information [1]. Our analysis revealed that, 20,792 unigenes (78.79 %) were mapped to the genome scaffolds, while 14,170 of the mapped (68.15 %) were similar to the predicted genes from the genome. Also, 5812 (50.12 %) of the protein coding genes predicted from the genome assembly were overlapped with the unigenes mapped against the draft genome. Moreover, 5289 (14.2 %) of the unigenes are non over lapping with the genome scaffolds and at an average of 2.438, more than one contig mapped to the same gene model. Since, ESTs are already available for *Spodoptera frugiperda* from different tissue/cell types, to attain confidence in the transcriptome, the assembled contigs were compared against the ESTs in SPODOBASE [7]. The analysis showed that, over 53 % of total ESTs aligned to the Sf21 transcripts, while over 60 % of the ESTs from *S. frugiperda* were aligned to the assembled contigs. These analyses confirmed that, the present transcriptome assembly is in conjunction with existing data of the genome as well the trascriptome [1, 7] and promises the improvement of genome scaffolds with further sequencing of higher read lengths.

In addition, length distribution of transcripts against the whole transcriptome revealed that, the contigs of length > 1Kbp cover over 87 % of the transcriptome, while the contigs of length 1-10Kbp cover ~ 82 % of the whole transcriptome (see Additional file 3B). Further, the sequence accuracy of the unigenes was examined using RT-PCR and Sanger sequencing. A total of 12 unigenes, such as GAPDH, β actin, α tubulin, rRNA and the factors involved in RNA silencing [8]. All the RT-PCR reactions produced specific amplicons, suggesting the primer specificity. The amplicons were further sequenced and the sequences were aligned to the unigene sequences with complete identity and no insertion or deletion. These results clearly indicate a good quality transcriptome, in particular, the assembly of identified unigenes.

Later, the analysis of nucleotide composition of the whole transcriptome revealed that, the mean GC content stood at 39.82 % similar to its levels in the draft genome assembly, which is 32.97 % [1]. Also, as shown in Additional file 4A, over 78 % of the transcripts lie in the GC range of 35–40 %, while similar levels were reported for its genome (26–40 %) [1] as well, indicating a closer molecular signature between the draft genome and the transcriptome of Sf21 cells. In addition, we measured the %GC in the transcriptome of relatively close insects, such as, *B. mori* [9]*, D. plexippus* [10]. As shown in Additional file 4B, in case of both *S. frugiperda* and *B. mori*, over 50 % of the transcripts lie in the GC range of 30–45 % while an over 30 % of the transcripts in *B. mori* extend their GC range to 55 %. But, the transcripts from *D. plexippus* showed an extended GC range of 40–55 % which is similar to *S. frugiperda* at the range of 40–45 % but, relatively following the pattern of *B. mori* all along, suggesting a pattern in the molecular signatures of coding between insect species, which are evolutionarily distant to each other.

The transcript/EST based markers are important resource for determining functional genetic variation [11]. Among the various molecular markers, SSRs are highly polymorphic, easier to develop and serve as rich source of diversity [12]. To identify the SSRs in Sf21 trancriptome, the tool, Mreps [13] was employed. The analyses revealed that, a total of 7867 transcripts contain the SSRs (2–6 nt), among which 2826 transcripts contain more than one SSR. A total of 12,704 SSRs (2–6 nt) identified with a frequency of one per 133 bp. Among the different classes of SSRs (2–6 nt) identified, the tri- and hexa- nucleotide occupy 49.65 % followed by tetra- (25.58 %) and penta- nucleotide (16.16 %) while the least present are di-nucleotide (8.59 %) (see Additional file 2B). However, the transcripts encode SSRs of higher in length (>6 nt) and the complete list of SSRs with their frequency and respective sequences are provided in Additional file 5. These observations coincide with previous reports suggesting a higher number of tri- and hexa- nucleotide SSRs when compared to the other categories in EST sequences.

The digital expression profiling, also called RNA-Seq is a powerful and efficient approach for gene expression analysis [14, 15]. The abundance information is useful for understanding the importance of identified genes. Relative abundance of the assembled unigenes was calculated using the tool, RSEM [16, 17]. The short reads were aligned back onto the assembled transcripts and the analysis revealed that, 97.76 % of all the transcripts were quantified with mean coverage and insert length of 348.8 and 257.74 respectively. Here, the abundance/quantification of unigenes were measured in terms of fragments per kilo base per million (FPKM). The FPKM values for the transcripts ranged from 0.09 to 27161.63, indicating a very wide range of expression levels of Sf21 transcripts (see Additional file 6). It also indicates that, even very low expressed transcripts were represented in our assembly. The relationship between the length of unigene and the FPKM values is presented in Additional file 7 and the Additional file 2C lists the most abundant transcripts (excluding the ribosomal proteins) in the Sf21 transcriptome. The transcripts annotated against the NCBI non redundant protein database indicated that, they belong to the cytochrome family and the translation machinery along with the heat shock proteins (see Additional file 6). This data would be of greater importance to further studies on physiological roles of the genes in the insect.

To annotate the unigenes, the transcripts were initially aligned against the UniProt insect protein data base and the un-annotated from the initial phase were later aligned against the NCBI invertebrate protein database. The analysis resulted in annotation of 8835 unigenes, which were then categorized into their respective Gene Ontology (GO) terms based on the annotation. Among the annotated having GO terms, ~ 49.61 % of them are under the category, Biological Process followed by Molecular Function (37.51 %) and Cellular Component (12.86 %) (see Additional file 8). In particular, the highly expressed ones are nucleotide binding, membrane component along with ATP binding, integral to membrane, intracellular component and nucleic acid binding (see Additional file 9). However, during annotation, over 60.8 % of the annotated genes showed homology with *B. mori* followed by *T. castenum* (11.61 %) and *D. melanogaster* (6.53 %), indicating a common selection of genes between closely related insect genuses.

In addition to the annotation based on homology, we assigned functional characteristics to the genes using ortholog gene clusters from NCBI KOG database. The analysis revealed that, over 36.6 % of the unigenes were assigned a functional characteristic based on the sequence information from the orthologs. Among them, over 77.5 % are assigned to a unique KOG characteristic and the proteins present in higher number are the ones involved in posttranslational modification, protein turnover and chaperones (see Additional file 10), besides the categories, General Function prediction and Function unknown. Also, we observed that, the unigenes share majority of KOGs are *Drosophila* (~39 %) followed by *H. sapiens* (26.34 %), *C. elegans* (13.54 %) and to the least in *S. cerevisiae* (6.69 %), *S. pombe* (3.23 %) and *E. cuniculi* (0.4 %) (see Additional file 11)*.*

Further, we identified the protein domain families using InterProScan [18]. The analysis revealed that, majority of genes possess the domains, Zinc finger C2H2 followed by Zinc finger C2H2-type/integrase DNA binding (see Additional files 12 and 13), suggesting a probable role for these proteins in survival and propagation of Sf21 cells. After the annotation through different modules, the unigenes were categorized into KEGG pathways based on their association. Among the enriched pathways for the genes are metabolic pathways and biosynthesis of secondary metabolites (see Additional file 3D) indicating the coding pattern of certain proteins necessary for the metabolism and survival of the insect, *Spodoptera frugiperda.* As a whole, the present analysis, in conjunction with the genome data, would provide a platform for delineating intron-exon structure among other features such as, transposon footprints, genes without apparent paralogs and coding signatures of gene sequences. Also, the data presented here would provide resources for functional genomics of *S. frugiperda* and investigation of mechanisms underlying the biology of the insect.

### Availability of data

The sequence reads and assembled contigs of the transcriptome from *Spodoptera frugiperda* cell line Sf21 is available at NCBI with accession no: SRX952269 and GCTM00000000 respectively.

Footnotes: The tools and methods employed in the present study are described in the Additional file 14: Materials and methods.

## Review, Round#1

### Response to reviewer comments

We thank the editor and the reviewers for their valuable comments/suggestions. We have carefully considered their suggestions and revised the manuscript. We appreciate reviewer suggestions to improve quality of transcriptome by seeking clarification on few descriptions of experiments. Specifically, reviewer#1 suggested analysis of data employing additional software Mreps. We have subjected our data to the suggested software. Results of the analysis by the suggested software have been incorporated in the revised version of the manuscript. Please find below the responses to each comment raised by both the reviewers;

### Reviewer#1, Dr Thiago Motta Venancio

Q: The S. frugiperda genome has been sequenced by the same group, who predicted ~12,000 genes. In the present work they report 26,390 unigenes, which is more than twice the number of genes reported in the genome paper. Since the authors have clustered the contigs, we can rule out most alternative splicing events. I think the number of genes predicted in the genome is closer to what would be expected for an insect. Authors should provide an explanation for such large discrepancy in the manuscript.

A: Yes, we share the reviewer’s concern. The Sf21 genome assembly is a draft in nature and the predicted genes reported only encode proteins. Since, the library made for transcriptome assembly is a poly(A) rich, the unigenes reported in the present study encodes both proteins as well other functional non coding RNAs. Considering the small length of sequenced reads used for the assembly, though clustering is done for predicted ORFs, we believe, there are still gaps and sequences that are truncated lead to incomplete/partial unigene sequences which could be part of a single gene. However, we are in the process of improving the contig length to have a comprehensive list of unigenes, which would make a basis for further communications.

Q: How can the number of contigs be lower than the number of unigenes?

A: The final Sf21 Transcriptome assembly contains only 24,038 contigs/transcripts. During the process, 80 k ORFs were identified and clustered to produce a set of 26,390 unigenes. Therefore, the larger transcripts encoding multiple ORFs lead to the higher number of unigenes.

Q: I am also surprised by the restricted use of the sequenced genome. Why have the authors performed a de novo assembly when a reference genome is available? Assembling de novo is really not the best alternative on the table. Mapping reads to the genome would be the best approach to find expression patterns along the genome and discover new protein-coding loci that might have been missed in the genome sequencing project.

A: Reviewer concern has been considered and our response is as follows. The reasons behind the use of de novo approach for the transcriptome assembly are listed below;

- The genome and transcriptome assembly were run in parallel, thus, it was not possible to make use of genome assembly for constructing the transcriptome.
- The published genome is still at draft / scaffold level and contains ~37 k sequences; which itself reflects the fragmentation level of the genome; thus not suitable for reference guided assembly.
- Further, the transcriptome reads would have to be mapped on to huge number of sequences and multi-mapping of reads could easily bias the transcript structure and expression estimation as the mapping software would treat them as genomic repeats.
- Though, we predicted gene models on the draft/fragmented/incomplete genome sequences, using a nearest homologous gene model (of *Bombyx mori*); these models are still being curated and hence subject to finer re interpretation.

Q: The authors used Spodobase to evaluate the quality of the transcriptome. I have a few questions/comments regarding this analysis:

1. When was the last update of this DB?

A: The database was last updated in July 2013 and the updated dataset was considered during the analysis.

2) Simply downloading the most complete set of ESTs from Genbank seems to be a better alternative;

A: Our response. As per SOPDOBASRE, “ESTs have been sorted either as singlet (86786) or within clusters (14654). Sequences belonging to clusters were assembled into consensus sequence called contigs, some clusters giving rise to several contigs. Sequences were compared against several databases: NCBI nr, *Bombyx mori* dbEST and Uniprot”. The reviewer may please refer the link, http://bioweb.ensam.inra.fr/Spodopterav3/browser for a better understanding.

3) Having 60 % of the ESTs from a database mapping to the transcriptome of a single cell type indicates that the database composition is biased towards the same or similar cell types;

A: No, the database composition is not biased towards any similar cell types. Infact, the ESTs deposited in Spodobase were sourced from *S. frugiperda* insect tissues like hemocytes, midgut and fat body and even the Sf9 cell lines. The reviewer may please refer the link, http://bioweb.ensam.inra.fr/Spodopterav3/browser for more information.

Q: Authors used MISA to identify SSRs. The higher prevalence of tetra-nucleotide SSRs is really unusual because SSRs in coding regions are of the tri- and hexa-nucleotide classes because they can keep the reading frame of the transcript intact. A tetra-nucleotide would result in a frameshift that would probably compromise the functions of the encoded protein. I am not familiar with MISA, but the use of a tool that finds maximal SSRs, such as mreps (PMID: 12824391), could give different results.

A: Yes, we agree with the reviewer’s concern. As suggested by the reviewer, the analysis was performed again employing the tool, Mreps and the results are incorporated in the modified version of the manuscript, page# 4, line#5 (Additional files 2B and 5).

Briefly,

…… a total of 7867 transcripts contain the SSRs (2–6 nt), among which 2826 transcripts contain more than one SSR. A total of 12,704 SSRs (2–6 nt) identified with a frequency of one per 133 bp. Among the different classes of SSRs (2–6 nt) identified, the tri- and hexa- nucleotide occupy 49.65 % followed by tetra- (25.58 %) and penta- nucleotide (16.16 %) while, the least present are di-nucleotide (8.59 %) (Additional file 7B). However, the transcripts encode SSRs of higher in length (>6 nt) and the complete list of SSRs with their frequency and respective sequences are provided in Additional file 9.

Q: The raw transcriptomic data should be made available at a public repository.

A: Yes, we agree with the reviewer’s comment. The raw data of the transcriptome was already available at NCBI SRA accession: SRX952269. Also, the assembled contigs were deposited in NCBI with accession no: GCTM00000000 and the data would be public soon the curation process completes from the NCBI staff. The same was mentioned in the manuscript text, page# 6.

Q: Some symbols were lost during PDF conversion.

A: Yes, we thank the reviewer for pointing out the error. It has been rectified in the revised version of the manuscript.

Q: Replace “coding for proteins” for “encoding proteins.

A: Yes, the suggestion has been incorporated in the revised version of the manuscript text.

### Reviewer#2, Professor Michael Gray

Q: The paper would benefit by some comment as to how the transcriptome data enhance the information obtained by a previously published draft genome sequence from the same group. An example would be the use of transcriptome data to elucidate the exon-intron structure of the genome. Although this aspect was commented on in the genome paper, a brief summary here would be helpful for readers of this paper, since comparison with genomic data is an obvious thing to do with transcriptome data.

A: Yes, we agree with the reviewer’s concern. A brief statement on the usefulness of the present transcriptome data in conjunction with the previously published genome data has been incorporated in the revised version of the manuscript, page# 5.

## Review, Round #2

### Response to reviewer comments

We thank the editor and the reviewers for their valuable comments/suggestions. We have carefully considered their suggestions and revised the manuscript. We appreciate reviewer suggestions to improve quality of transcriptome by seeking clarification on the data sets we employed. Specifically, reviewer#1 suggested comparison of the transcriptome data with the genome information. We performed the suggested analysis and the results have been incorporated in the revised version of the manuscript. Please find below the responses to each comment raised by both the reviewers;

### Reviewer#1, Dr Thiago Motta Venancio

Q: The authors argue that the transcriptome was assembled de novo and the reads not mapped to the reference genome because the projects have been conducted in parallel. Nevertheless, this study warrants a clear some connection to the genome paper. I would recommend the authors to simply map the contigs or unigenes to the predicted genes to answer basic questions like: 1) How many predicted genes can be detected in the transcriptome? 2) How many new genes could be predicted with the transcriptome data (i.e. those mapping to loci without gene predictions) ? 3) How often do more than one contig map to the same gene model? My main concern with the lack of a clear comparison between the genome and the transcriptome is that the former, at least in terms of size, is closer to what I would expect for a lepidopteran genome. Therefore, I suspect the transcriptome is still very fragmented and the community would benefit from some basic comparisons to have a clearer picture of what can be concluded by the genome and transcriptome sequencing projects together.

A: We considered the reviewer comment and performed a BLAT analysis with 70 % coverage and identity by comparing the transcriptome data against the genome information. Our analysis revealed that, 20,792 unigenes (78.79 %) were mapped to the genome scaffolds, while 14,170 of the mapped (68.15 %) were similar to the predicted genes from the genome. Also, 5812 (50.12 %) of the protein coding genes predicted from the genome assembly were overlapped with the unigenes mapped against the draft genome. Moreover, at an average of 2.438, more than one contig mapped to the same gene model. Further, 5289 (14.2 %) of the unigenes are non over lapping with the genome scaffolds, promising the improvement of genome scaffolds with further sequencing of higher read lengths. This data has now been incorporated in the revised version of the manuscript text, page#3, line#6.

Q: Authors argue that the Spodobase DB is not biased in terms of sequence source. However, 60 % of all its reads map to the transcriptome assembly presented here, which was derived from a single cell type. Having more than one cell type in the database does not mean it is not biased. To show that the database is not biased one needs to check its sequence distribution across different cell types.

A: We considered the reviewer comment. Please find below the EST distribution in SPODOBASE from different tissue/cell types of *Spodoptera frugiperda.*

**Table:** 

**CODE**	**Count**	**%**	**Tissue type**
Sf1F	7171	3.68	Fat body
Sf1H	6000	3.08	Hemocyte
Sf1M	6149	3.15	Midgut
Sf1P	28928	14.83	Pool of various tissues
Sf2H	9686	4.97	Immune Challenged hemocytes
Sf2L	2366	1.21	Sf21 Cell lines sequences from R. CLEM
Sf2M	13026	6.68	Xenobiotic Induced Midgut
SF9L	5822	2.99	Sf9 cell lines sequences
Sf9LR	115862	59.41	Sf9 cell line from G. Rohrmann

The table shows that other cell types have also been considered. However, more specific cell type based transcriptome data needs to be generated to make meaningful comparison.

Q: In addition to the raw sequences the community also needs access to the assembly itself. I would recommend the TSA database for this purpose: http://www.ncbi.nlm.nih.gov/genbank/tsa

A: Yes, we understand the reviewer concern. As mentioned in the manuscript text, page#6, last paragraph, the assembled transcripts were already deposited in NCBI TSA database and assigned the accession no: GCTM00000000. As soon the NCBI staff curate the data, it would be released to the public domain.

Minor points:

Q: Update flowchart to include mreps.

A: Agreed. The flowchart was modified to include mreps in the revised version.

Q: Replace “frame” by “open reading frame”.

A: Agreed. The word, “frame” was replaced by “open reading frame” in the revised manuscript text.

Q: Some symbols remain corrupted in the PDF.

A: Corrected

### Reviewer#2, Professor Michael Gray

Q: There are still a few symbols that have not been rendered correctly in the PDF of the revised manuscript (e.g., pg. 3, line 11, should read “ ~ 82 %”), so the authors should take note of this issue.

A: Corrected.

## Additional files

Additional file 1:
**Statistics of the Trinity and Velvet-Oasis assemblies.** (XLS 1460 kb) Additional file 2:
**(A) Summary of statistics from final transcripts of Sf21 cells.** (B) Statistics of SSRs identified from Sf21 transcripts. (C) The top 10 most abundant unigenes identified. (D) The top KEGG pathways of the identified unigenes. (PDF 110 kb) Additional file 3:
**Distribution of transcripts and unigenes: (A) Length wise distribution of the unigenes identified from the Sf21 transcriptome.** (B) Length wise distribution of individual transcripts and their share in the whole transcriptome. (TIFF 1970 kb) Additional file 4:
**Analysis of GC content: (A) Comparison of GC content from both the transcriptome and draft genome of Sf21 cells.** (B) Comparison of GC content from Sf21 transcripts with other lepidopteran insects, *B. mori* and *D. plexippus.* (TIFF 1992 kb) Additional file 5:
**Complete list of SSRs identified in the transcripts.** (XLS 4963 kb) Additional file 6:
**Complete list of abundance of the identified unigenes.** (XLS 3495 kb) Additional file 7:
**Abundance of identified unigenes: Abundance of the identified unigenes versus their respective lengths in terms of FPKM value.** (TIFF 1633 kb) Additional file 8:
**Gene Ontology (GO) analysis of the annotated unigenes.** (XLS 3759 kb) Additional file 9:
**Gene Ontology classification of the identified unigenes: Gene ontology (GO) term was assigned to each gene based on the annotation and were summarized into three main GO categories (biological process, cellular component, molecular function) while only the top 10 sub categories are presented in individual pie charts.** (TIFF 1286 kb) Additional file 10:
**COG functional classification of the unigenes: The functional characteristic assigned to the unigenes through homology against NCBI COG database and their classification among the 25 categories.** (TIFF 3364 kb) Additional file 11:
**KOG analysis of the identified unigenes**. (XLS 1321 kb) Additional file 12:
**Analysis of protein domains in the unigenes: Distribution of protein families/domains among the genes identified from the transcriptome.** The twenty most abundant families are presented with their respective family size. (TIFF 2785 kb) Additional file 13:
**Protein domain analysis of the identified unigenes.** (XLS 7898 kb) Additional file 14:
**Materials and methods.** (DOC 28 kb)

## Abbreviations

Sf: *Spodoptera frugiperda*
Mb: Million bases
Kbp: Kilo base pair
EST: Expressed Sequence Tag
cDNA: Complimentary DNA
SSR: Simple Sequence Repeat
FPKM: Fragments Per Kilo base per Million
GO: Gene Ontology
PCR: Polymerase Chain Reaction
RT-PCR: Reverse Transcriptase- Polymerase Chain Reaction
qRT-PCR: Quantitative Reverse Transcriptase- Polymerase Chain Reaction
