# Supplementary figures and images for: De novo transcriptome assembly and analysis of Sf21 cells using illumina paired end sequencing
Source: Biol Direct. 2015 Aug 20;10:44. doi: 10.1186/s13062-015-0072-7 (PMC4545970; doi:10.1186/s13062-015-0072-7)

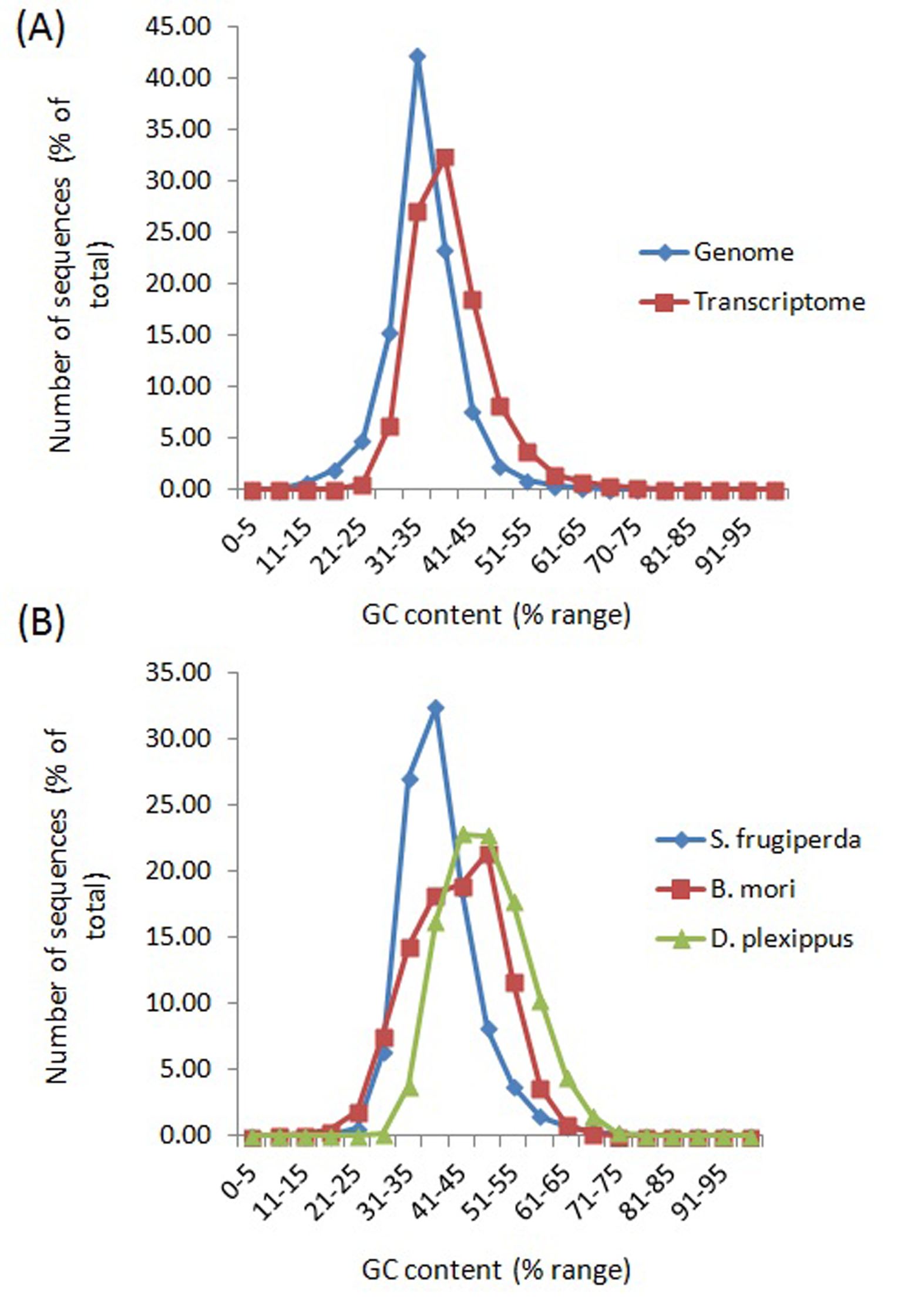

Supplement: Additional file 1: — Statistics of the Trinity and Velvet-Oasis assemblies. (XLS 1460 kb) [file 13062_2015_72_MOESM1_ESM.tiff]

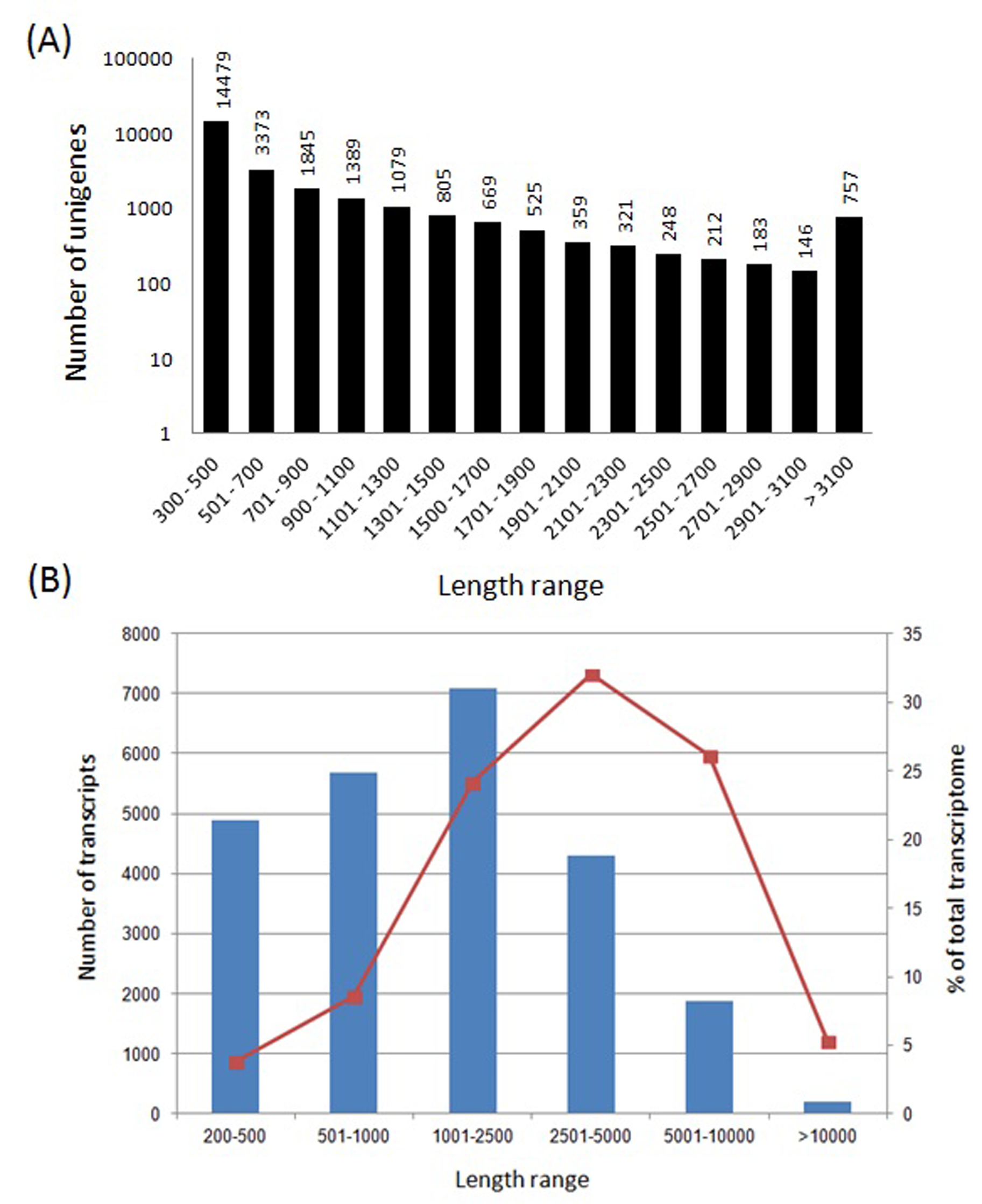

Supplement: Additional file 2: — (A) Summary of statistics from final transcripts of Sf21 cells. (B) Statistics of SSRs identified from Sf21 transcripts. (C) The top 10 most abundant unigenes identified. (D) The top KEGG pathways of the identified unigenes. (PDF 110 kb) [file 13062_2015_72_MOESM2_ESM.tiff]

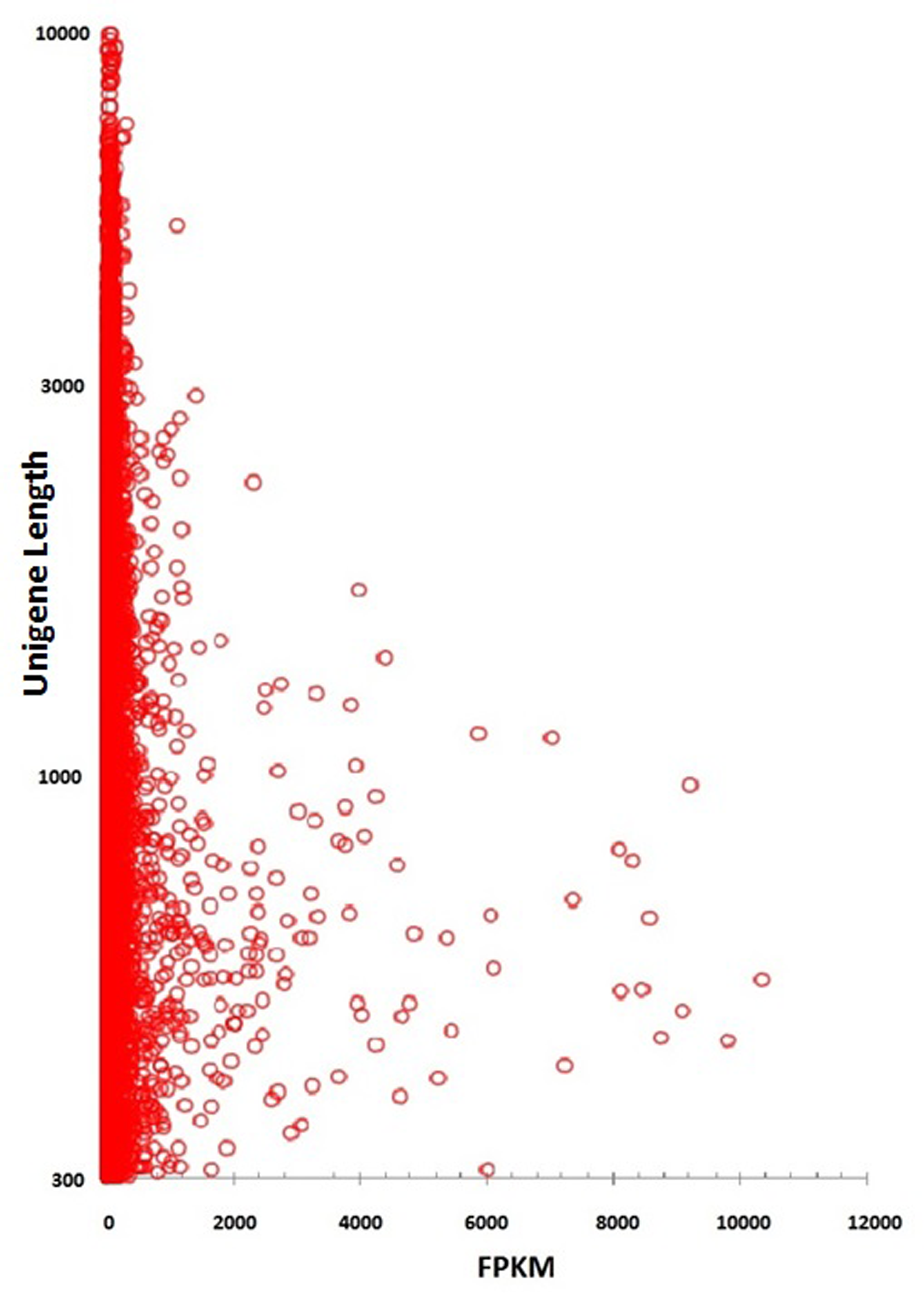

Supplement: Additional file 3: — Distribution of transcripts and unigenes: (A) Length wise distribution of the unigenes identified from the Sf21 transcriptome. (B) Length wise distribution of individual transcripts and their share in the whole transcriptome. (TIFF 1970 kb) [file 13062_2015_72_MOESM3_ESM.tiff]

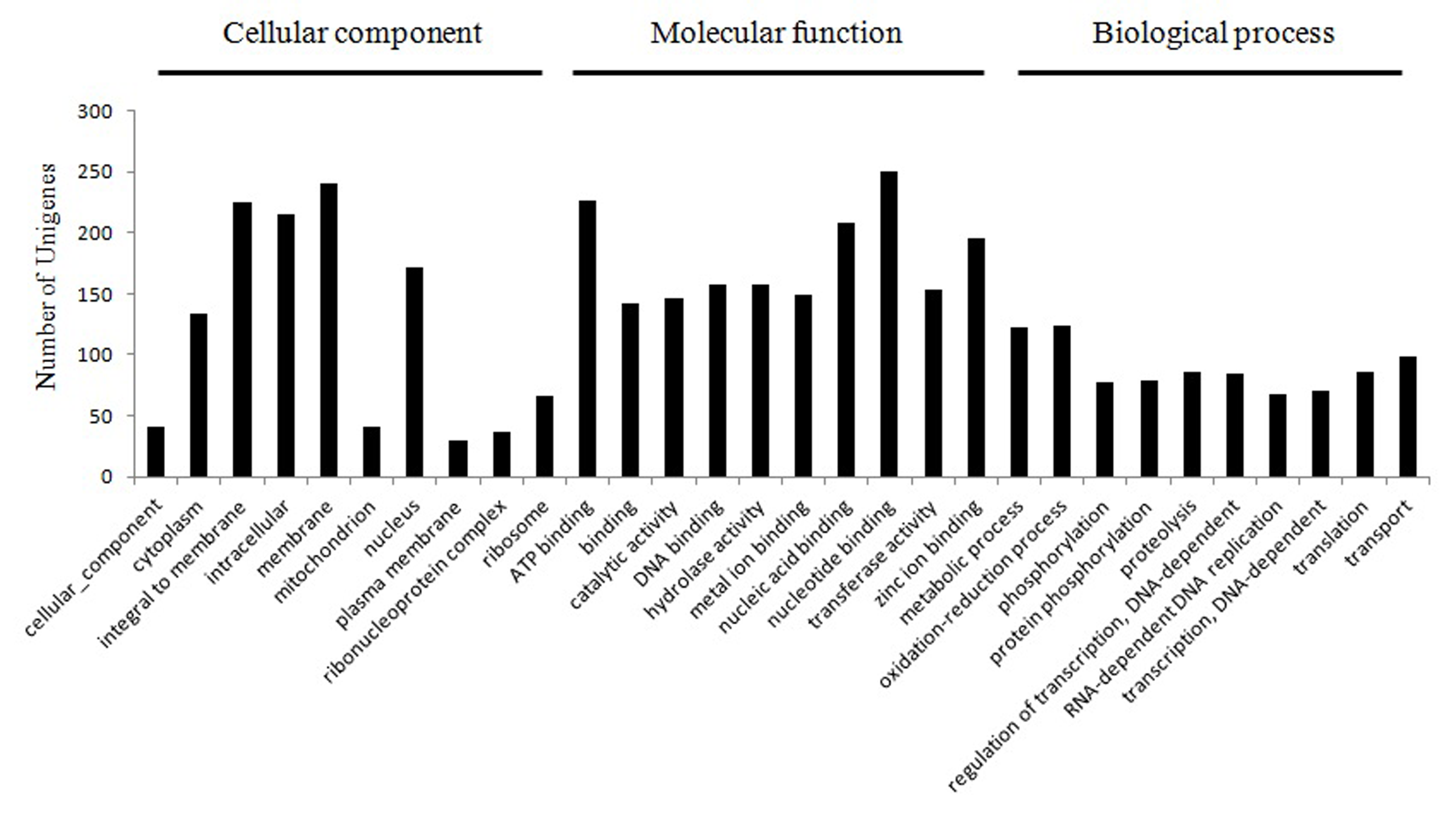

Supplement: Additional file 4: — Analysis of GC content: (A) Comparison of GC content from both the transcriptome and draft genome of Sf21 cells. (B) Comparison of GC content from Sf21 transcripts with other lepidopteran insects, B. mori and D. plexippus. (TIFF 1992 kb) [file 13062_2015_72_MOESM4_ESM.tiff]

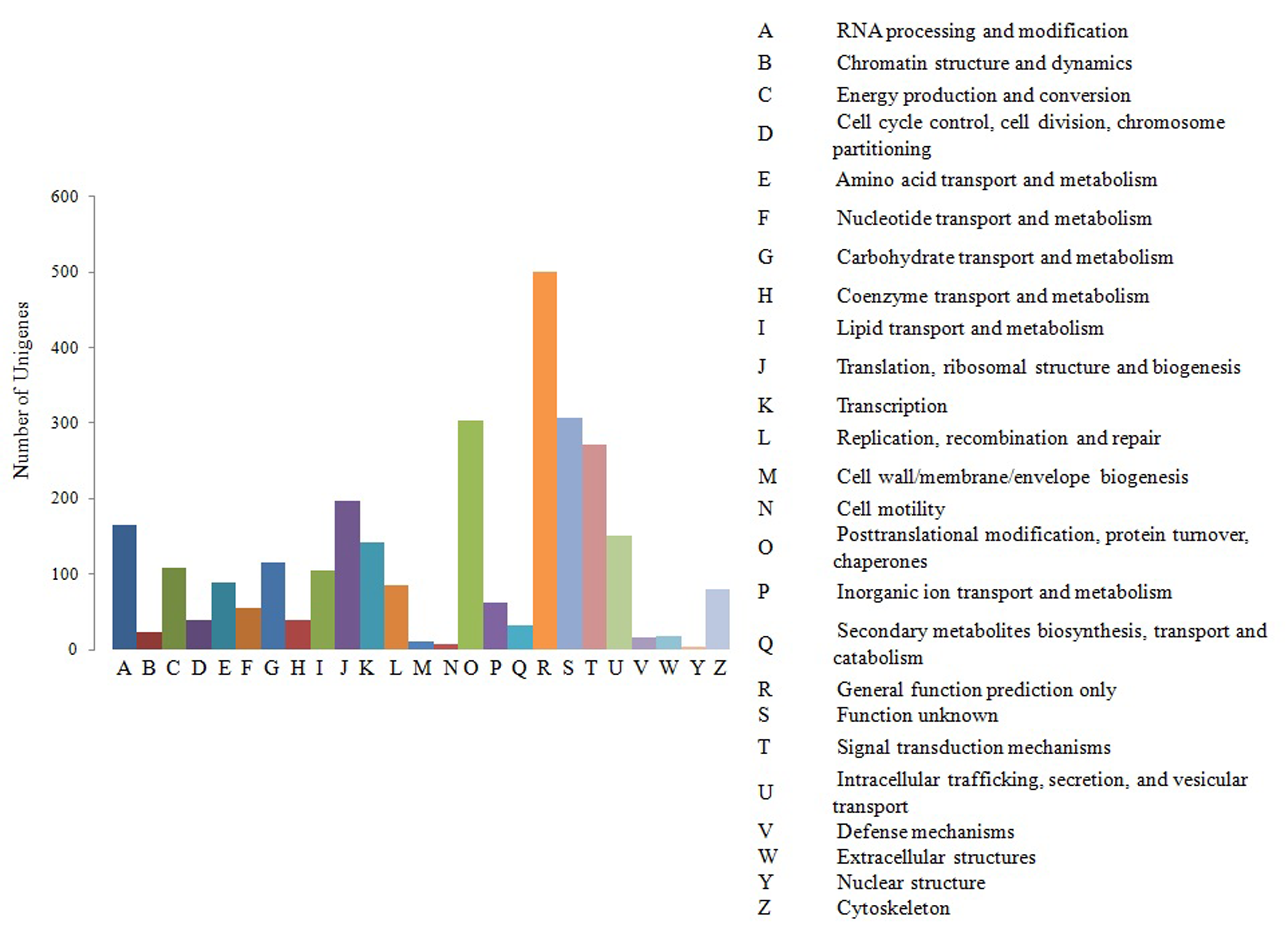

Supplement: Additional file 5: — Complete list of SSRs identified in the transcripts. (XLS 4963 kb) [file 13062_2015_72_MOESM5_ESM.tiff]

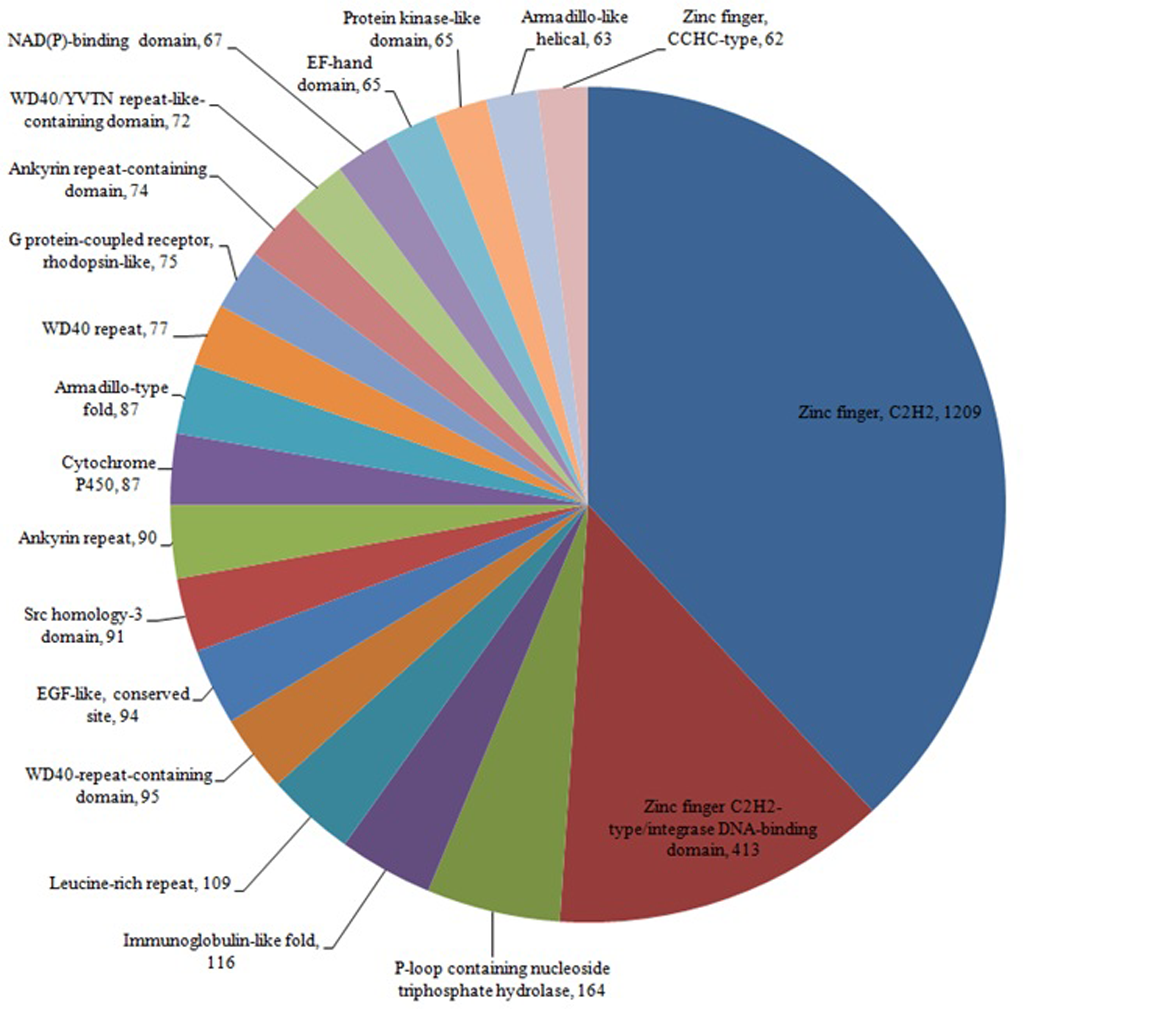

Supplement: Additional file 6: — Complete list of abundance of the identified unigenes. (XLS 3495 kb) [file 13062_2015_72_MOESM6_ESM.tiff]
