# Supplementary material for: De novo transcriptome assembly and analysis of Sf21 cells using illumina paired end sequencing
Source: Biol Direct. 2015 Aug 20;10:44. doi: 10.1186/s13062-015-0072-7 (PMC4545970; doi:10.1186/s13062-015-0072-7)
Supplement: Additional file 7: — Abundance of identified unigenes: Abundance of the identified unigenes versus their respective lengths in terms of FPKM value. (TIFF 1633 kb) [file 13062_2015_72_MOESM7_ESM.pdf]

(A)

Total number of contigs	24038
Length (Mb)	47.38
Mean	1971.11 bp
Median	1199 bp
Min	201 bp
Max	28914 bp
N10	8293
N20	6342
N30	5139
N40	4224
N50	3409
N60	2745
N70	2056
N80	1452
N90	854

(B)

**SSR mining**	
Number of sequences examined	24038
Total length of the sequences	47381605bp
No. of SSRs identified	2229
No. of sequences containing SSRs	1898
No. of sequences containing more than one SSR	260
No. of SSRs present in compound formation	78
Frequency of SSRs	One per 21.25 Kb
**Distribution of SSRs in different repeat types**	
Di-nucleotide	575
Tri-nucleotide	740
Tetra-nucleotide	791
Penta-nucleotide	83
Hexa-nucleotide	41

(C)

ORF ID	Length	FPKM	Blast Hit	% Identity	E value
**SF21_Tr11603_3_[488_-_1858]**	1371	2744.7	Eukaryotic translation initiation factor 4A	96.44	0
**SF21_Tr2887_6_[4524_-_3196]_R_Sense)**	1329	3309.77	S-adenosylmethionine synthetase	96.29	0
**SF21_Tr15952_12_[2501_-_1668]_R_Sense)**	834	3750.49	Receptor for activated protein kinase C (Receptor for activated protein kinase C RACK isoform 1)	97.56	0
**SF21_Tr1228_6_[2397_-_4226]**	1830	3947.29	Chorion b-ZIP transcription factor	50.65	2.00E-143
**SF21_Tr11937_1_[366_-_1223]**	858	4052.25	Cytochrome b (Fragment)	84	3.00E-105
**SF21_Tr6204_18_[10366_-_8882]_R_Sense)**	1485	4365.09	AT07372p (Heat shock protein cognate 1, isoform C) (EC 3.6.1.3) (Heat shock protein cognate 1, isoform D) (EC 3.6.1.3)	89.69	0
**SF21_Tr16332_2_[570_-_55]_R_Sense)**	516	4757.69	Cytochrome c oxidase subunit 2	86.99	2.00E-062
**SF21_Tr1856_2_[3578_-_4207]**	630	5353.72	Eukaryotic initiation factor 5A (Eukaryotic translation initiation factor 5A) (Translation initiation factor 5A) (Uncharacterized protein)	98.75	2.00E-116
**SF21_Tr6648_3_[41_-_1219]**	1179	5848.17	ADP/ATP translocase	97.47	0
**SF21_Tr732_3_[1184_-_24]_R_Sense)**	1161	7027.45	Cytochrome c oxidase subunit 1 (EC 1.9.3.1) (Fragment)	85.68	0

(D)

ko_ID	Pathway_Name	No. of Unigenes
ko01100	Metabolic pathways	77
ko01110	Biosynthesis of secondary metabolites	19
ko04144	Endocytosis	18
ko04120	Ubiquitin mediated proteolysis	16
ko03040	Spliceosome	15
ko04141	Protein processing in endoplasmic reticulum	15
ko03010	Ribosome	14
ko00230	Purine metabolism	13
ko04810	Regulation of actin cytoskeleton	13
ko04510	Focal adhesion	13
