# Supplementary material for: De novo transcriptome assembly and analysis of Sf21 cells using illumina paired end sequencing
Source: Biol Direct. 2015 Aug 20;10:44. doi: 10.1186/s13062-015-0072-7 (PMC4545970; doi:10.1186/s13062-015-0072-7)
Supplement: Additional file 14: — Materials and methods. (DOC 28 kb) [file 13062_2015_72_MOESM14_ESM.doc]

**Materials and methods**

**Samples and RNA extraction**

The Sf21 cell line were purchased from Life Technologies, USA and maintained in TNM-FH medium at 27°C in a humidified incubator. Total RNA was isolated from the cell lines using Trizol (Life Technologies, USA) as per manufacturer instructions. The content of total
RNA was determined using the NanoDrop 2000 Spectrophotometer (Thermo Scientific, USA) and the quality was assessed by agarose gel electrophoresis.

**Sequencing and *de novo* assembly**

The total RNA isolated from *Sf*21 cells was initially run for a quality check and then sequenced on Illumina HiSeq2000 platform (San Diego, California, USA) with a paired end fragment length of 400bp. The raw reads were filtered, obtaining a high quality (HQ) reads to generate the assembly using Trinity [7] and Velvet [8]. The transcripts were next clustered CD-HIT-EST tool [9] to generate non redundant contigs.

**Quality assessment of the assembly**

To assess the quality of the assembly, the contigs were aligned against the EST data in SPODOBASE [4] using BLASTN. In addition, 12 unigenes homologous to known proteins were amplified from the total RNA, using Qiagen One Step RT-PCR kit (Qiagen, USA) using respective primers of each gene. The sequences of the primers used in the study are presented in Table S1.

**Abundance estimation**

The relative abundance of assembled unigenes was calculated using RSEM [10]. The raw reads were first aligned to the unigenes generated from the non redundant contigs with single-end read mode. Next, the relative abundance of each unigene expressed as FPKM, calculated from the alignment results.

**Identification of EST-SSRs**

Potential EST-SSR markers were detected within the assembled transcripts using the microsatellite identification tool, MISA [11]. The parameters were adjusted for identification of di-, tri-, tetra-, penta-, and hexanucleotide motifs while ignoring the mononucleotide repeats.

**Functional annotation**

The assembled unigenes were searched against UniProt insect protein data base and NCBI invertebrate protein database. Using BLASTX, the unigenes were annotated, satisfying the set criteria and categorized into respective Gene Ontology (GO) terms. Further, to identify functional characteristics, the predicted genes were aligned against the NCBI KOG database using BLASTN. In addition, InterProScan [12] database was used to screen for protein families from the unigene list.
